# Catastrophic expenditure associated with childhood hospitalisation for acute illness in Kenya and Uganda: a cross-sectional study

**DOI:** 10.1136/bmjph-2024-001173

**Published:** 2025-01-16

**Authors:** Julie Jemutai, Rebecca Gathoni Njuguna, Yoko V Laurence, Sheila Murunga, Caroline Ogwang, Shalton Mwaringa, Laura Mwalekwa, Molline Timbwa, George Paasi, Peter Olupot-Olupot, Mwanajuma Juma, Johnstone Thitiri, Narshion Ngao, Benson Singa, John Mukisa, Ezekiel Mupere, Christina Lancioni, Priya Sukhtankar, Kirkby D Tickell, Md Fakhar Uddin, Sassy Molyneux, Judd L Walson, Anna Vassall, James Berkley

**Affiliations:** 1 Health Systems and Research Ethics Department, KEMRI-Wellcome Trust Research Programme, Kilifi, Kenya; 2 Clinical Research Department, KEMRI-Wellcome Trust Research Programme, Kilifi, Kenya; 3 Childhood Acute Illness and Nutrition (CHAIN) Network, Nairobi, Kenya; 4 AkiliNook Research and Consulting, Kilifi, Kenya; 5 Nuffield Department of Population Health, University of Oxford, Oxford, UK; 6 Department of Global Health and Development, London School of Hygiene & Tropical Medicine, London, UK; 7 Health Economics for Life Sciences and Medicine, Department of Population Health Sciences, King's College London, London, UK; 8 Centre for Global Health, Kenya Medical Research Institute, Malaria Branch, Kisumu, Kenya; 9 Department of Public Health, Mbale Clinical Research Institute, Busitema University, Mbale, Uganda; 10 Kenya Medical Research Institute, Nairobi, Kenya; 11 Department of Immunology and Molecular Biology, Makerere University College of Health Sciences, Kampala, Uganda; 12 Department of Paediatrics and Child Health, School of Medicine College of Health Sciences, Makerere University, Kampala, Uganda; 13 Uganda Case Western Reserve University Research Collaboration, Upper Mulago Hill Road, Kampala, Uganda; 14 Department of Paediatrics, Oregon Health and Science University, Portland, Oregon, USA; 15 Department of Global Health, University of Washington, Seattle, Washington, USA; 16 Nutrition Research Division, International Centre for Diarrhoeal Disease Research (icddr,b), Dhaka, Bangladesh; 17 Centre for Tropical Medicine and Global Health, Nuffield Department of Clinical Medicine, Oxford University, Oxford, UK; 18 Department of International Health, Johns Hopkins Bloomberg School of Public Health, Baltimore, Maryland, USA

**Keywords:** economics, Nutrition Assessment, Cross-Sectional Studies, malnutrition, family

## Abstract

**Introduction:**

Childhood illness and hospitalisation result in both direct and indirect costs to families before, during and after admission. We aimed to estimate the catastrophic expenditure during hospitalisation for children with acute illness.

**Methods:**

This was a prespecified cross-sectional substudy nested within two prospective studies. Participants were recruited and interviewed from three rural and three urban hospitals in Kenya and Uganda. A costing questionnaire was administered to the caregivers of 731 children hospitalised for acute illness to evaluate direct and indirect costs incurred by caregivers and families. Costs incurred were compared for families with children both with and without complicated severe malnutrition (CSM). Catastrophic out-of-pocket expenditure exceeding 10% and 25% of monthly income was assessed.

**Results:**

The median (IQR) total cost during hospitalisation per child was US$47 (US$24–US$84), with higher costs for children with CSM, especially during hospitalisation (US$56 (US$26–US$99) vs US$36 (US$20–US$65); p<0.001). During hospitalisation, bed charges followed by food were the main cost drivers. Caregivers reported losing a median of 7 (4–11) days of productive time during a child’s hospitalisation with a mean loss of income of US$10 (SD US$25.6, median US$0 (US$0–US$10)). 92% and 74% of households experienced catastrophic expenditure at thresholds of 10% and 25% of monthly income, respectively. Caregivers reported borrowing, selling property and withdrawing other children from school to cope with costs.

**Conclusions:**

Despite intentions of free healthcare services for under 5, families of acutely ill children very commonly faced catastrophic expenditure, especially for children with CSM. Interventions aimed at supporting financial protection, reducing additional healthcare costs, and lowering health service charges may help prevent catastrophic expenditures.

**WHAT IS ALREADY KNOWN ON THIS TOPIC**

- Children hospitalised with acute illness incur significant costs from the health providers’ perspective.
- There is limited evidence of catastrophic expenditure faced by these children and their families during hospitalisation.

**WHAT THIS STUDY ADDS**

- When we interviewed caregivers from Kenya and Uganda, we found up to 90% of households experienced catastrophic expenditure due to hospitalisation of acutely ill children. The elevated risk of these costs was especially experienced by children with complicated severe malnutrition.

**HOW THIS STUDY MIGHT AFFECT RESEARCH, PRACTICE OR POLICY**

- To mitigate these costs, critical cost-effective and pro-poor interventions is paramount, especially for children under 5 years.
- Recognising vulnerability to, and protection from, these costs will require innovative social security measures by governments, non-government and community organisations.

## Background

 Despite declining global child mortality, >5 million children died in 2018.^1^ Low-income and middle-income countries (LMICs) bear the highest burden, with sub-Saharan Africa (sSA), having a child mortality rate 16 times greater than high-income countries.^1^

Within the context of the Sustainable Development Goals (SDGs), countries are committed to universal health coverage (UHC) and financial protection for households. However, health financing mechanisms in LMICs often rely on out-of-pocket (OOP) payments^2 3^ which can adversely impact health and nutrition, and further healthcare seeking.^4^ Even in countries that provisionally provide free services to under 5s, costs remain a major barrier, especially for the ~30%–40% of families living on less than US$2.15 per day in Kenya and Uganda.^5 6^ Thus, OOPs may hinder UHC^7^ and progress towards the SDGs.^8^

Healthcare for under 5s is intended to be free of charge in many sSA countries. However, facilities often charge hidden fees, such as registration costs or bed charges for caregivers who are required to stay with the child in hospital.^9^ Families commonly face other costs including transport, medicines, investigations, food, diapers, as well as losing income. Reported costs (including user charges, transport and medicine) vary by location, clinical syndrome and assessment method, with a range of US$8–US$74 per admission.^10^,^12^ Anticipated costs and loss of income may lead to families presenting their child hospital late, and/or leaving hospital without informing staff (absconding) or against medical advice,.^13^,^15^

Typically, 10%–25% of severely ill children admitted to hospital with common conditions including pneumonia, diarrhoea and febrile illness meet anthropometric criteria for severe malnutrition (complicated severe malnutrition, CSM).^16^ Both inpatient and postdischarge mortality in children under 5 is underpinned by CSM,^13^ which is both a significant medical condition and a proxy for other social difficulties. CSM is associated with poverty, chronic comorbidities, longer stays in hospital, prolonged attendance of clinics for therapeutic feeding and increased risks of readmission and postdischarge mortality.^13^ Reported costs incurred by families for inpatient treatment for a child with CSM range widely from US$0.5 to US$82^17 18^ without considering preadmission and postdischarge costs and income losses.

We aimed to assess direct and indirect costs associated with hospitalisation incurred by families of children admitted for acute illness with and without CSM in Kenya and Uganda, the potential economic impact of costs on households using measures of catastrophic expenditure and families’ mitigating or coping strategies.

## Methods

### Study design and participants

We conducted a prespecified cross-sectional cost-of-illness substudy nested within two prospective studies: the FLACSAM trial—ClinicalTrials.gov ID NCT03174236^19^ and the CHAIN cohort study.^13^ Caregivers were recruited at three rural and three urban hospitals in Kenya and Uganda: Kilifi County Hospital; Coast General Teaching & Referral Hospital, Mombasa; Mbagathi County Hospital, Nairobi; and Migori County Referral Hospital in Kenya; and Mulago National Referral Hospital, Kampala; and Mbale Regional Referral Hospital in Uganda.

The FLACSAM trial tested two inpatient antimicrobial strategies in a 2×2 factorial design among children aged 2 months to 13 years with CSM at admission to hospital, following them up at hospital discharge, days 14, 45 and 90 after enrolment (cohort A) (online supplemental etable 1). In addition, FLACSAM enrolled a parallel group of non-CSM children admitted to hospital with acute illness (cohort B) to investigate carriage of antimicrobial resistance and followed them up until hospital discharge. The CHAIN cohort enrolled acutely ill children aged 2–23 months at admission to hospital in three strata by nutritional status, including CSM and non-CSM (cohort C) and followed them up at days 45, 90 and 180 after discharge from hospital. We identified participants at discharge using consecutive sampling within the parent studies and invited caregivers to join the costing study. We excluded children who died during admission due to ethical concerns such as interviewing caregivers about costs when distressed.

Clinical management in hospitals followed WHO and national guidelines.^20^,^22^ Children were tested for HIV and referred to comprehensive care clinics as per national policy. Other comorbidities such as cerebral palsy, cardiac disease and haemoglobinopathies were investigated and treated as clinically indicated and referred to available services. At discharge, children with CSM were referred to an outpatient therapeutic feeding programme. The parent studies paid fares to attend non-study care study follow-up visits but not costs related to the index admission or outpatient care.

### Patient involvement

Participants in this study were not involved in the design, conduct, reporting or dissemination plans of the research.

### Data collection

The parent studies collected demographic, anthropometric, clinical, basic household characteristics, education and employment status of the caregiver at admission, and daily treatment received in hospital prospectively, using harmonised case report forms (CRFs).

In addition, we developed a structured patient cost questionnaire based on existing tools^23 24^ to assess costs during preadmission, from onset of symptoms to admission for the current illness, during hospitalisation and for 45 days postdischarge. Questions included direct healthcare costs, direct non-healthcare costs, indirect costs, mechanisms for paying costs, sources of payment, coping strategies and public or private health insurance. Caregivers were also asked to estimate costs incurred by any person accompanying them who contributed significantly to supporting the child or caregiver on admission and during hospitalisation, and costs relating to the care of other children or dependents incurred due to the caregiver being at the hospital. Self-report was used to estimate household and individual caregiver monthly income, while days lost were collected for a companion and any additional carers rather than their reported income because the information might not be known by caregivers. Household income was inclusive of caregiver’s individual income. The questionnaire also included details of household structure, assets and spending patterns. The tool was piloted among 22 caregivers in Kenyan and Ugandan hospitals between October and November 2017 and revised based on feedback. The final costing tool is available on the CHAIN Network website.^25^

The standardised costing tool was administered prospectively between March 2018 and December 2019 to caregivers at hospital discharge (cohorts A, B and C) and repeated at the day 45 follow-up (cohorts A and C only). Caregivers not able to physically attend this follow-up visit were interviewed by telephone.

### Statistical analysis

Data were captured on paper CRFs and entered in OpenClinica, V.3^26^ and REDCap^27 28^ with continuous data query resolution. Analyses were conducted in R V.4.0.3^29^ and STATA V.15. Costs were collected in Kenyan (KES) and Ugandan (UGX) Shillings, and converted to US dollars (USD) using the average rate for 2018/2019 (US$1=KES102=UGX3599).^30^

Our analysis was from the perspective of patients and their families to understand the financial burden of acute illness on them. A bottom-up microcosting approach was used to generate all the costs incurred by patients and their families where all relevant cost components were identified and valued. Prevalence-based approach was used to calculate the total costs of child acute illness for the assessed period. Direct costs were disaggregated into OOP healthcare costs and non-healthcare costs, covering preadmission, during hospitalisation and postdischarge. Direct healthcare costs included payments for consultation, registration, bed usage (for both child and caregiver), drugs, laboratory and radiology tests. Direct non-healthcare costs included transport to the hospital, food for child and caregiver, commercial diapers (most hospitals required them), childcare for other dependents, companion costs and other miscellaneous costs.

We reported proportions, mean and median costs where applicable with corresponding CIs, SD or IQRs. The total direct costs were derived by summing OOP healthcare and non-healthcare costs. Time horizon used for costing was in accordance with the time costing tools were administered, that is, hospitalisation costs were calculated from admission until discharge while postdischarge costs were costs incurred between discharge and 45 days after discharge.

Indirect costs due to time away from their normal business and household duties to access medical care were assumed to be equivalent to productivity loss. Time included travel and waiting time to access healthcare services. Losses from paid and unpaid work, as well as informal care, are key contributors to productivity loss. Informal care to sick family members has been shown to have detrimental effects on the caregiver’s overall health and similarly impose significant economic burden. The monetary valuation of productivity loss was assessed using individual reported income for income earners multiplied by the time lost in days. In order to determine household economic shock, we assessed the OOP payments as a proportion of monthly household income and defined catastrophic health spending as expenditure exceeding 10% and 25%.^31^,^34^

In some cases, obtaining data on self-reported income was challenging due to participants’ unwillingness to disclose the information or not knowing the full household income. We, therefore, also conducted analyses of the distribution of costs across social economic status groups using data on household assets and then derived a household asset score and tertiles to generate information on the relative household economic situation using principal component analysis (PCA).^35^ For missing socioeconomic data, imputation based on other demographic and socioeconomic features was performed using regularised iterative PCA algorithm (‘imputePCA’).^36^,^38^ Any extreme outliers were excluded from analysis.

We assessed the differences in direct and indirect costs preadmission and during hospitalisation between children with and without CSM. Due to skewed data, we applied a two-sample Wilcoxon rank-sum test and the Kruskal-Wallis test to compare the distributions between the two groups.

### Sensitivity analyses

Sensitivity analysis included scenario analysis to assess different methods for estimating indirect costs. In addition to calculating productivity costs based on caregiver’s reported lost income and days, we assessed reporting uncertainty of wage estimates for income earners and informal workers, as well as the imputation methods for non-income earners using expected minimum wage, gross domestic product (GDP) per capita and gross national income per capita and substituting forgone wage for non-income earners using informal workers’ wage rate. We applied different threshold values (5%, 10%, 15%, 20%, 25% and 30%) of catastrophic expenditure to illustrate the impacts on the proportion of households experiencing economic shock. We carried out sensitivity analyses of catastrophic expenditure using reported household income and imputed missing household income as derived from individual income.

## Results

### Participant characteristics

We interviewed 731 caregivers with median age of 28 years (IQR 23–34), of whom 436 (60%) had attained primary level education. More than half reported no sources of individual income (table 1). Children had a median age of 14 months (IQR 8–24) and 494 (68%) had CSM. Median reported monthly household income was US$78 (IQR US$28–US$155): US$69 (IQR US$20–US$147) for CSM and US$98 (IQR US$39–US$176) for non-CSM (p=0.05), respectively. Those with CSM had lower reported asset index compared with non-CSM (p=0.002). The median length of stay in hospital was 9 days for CSM and 4 days for non-CSM children (p<0.001).

**Table 1:** Participants' characteristics

Characteristics	CSMN=494	Non-CSMN=237
Study sites, n (%)		
Kilifi, Kenya	138 (28)	50 (21)
Mombasa, Kenya	130 (26)	72 (30)
Nairobi, Kenya	115 (23)	50 (21)
Migori, Kenya	23 (5)	18 (8)
Mbale, Uganda	67 (14)	28 (12)
Kampala, Uganda	21 (5)	19 (8)
Rural sites, n (%)	228 (46)	96 (41)
Child		
Female, n (%)	232 (47)	103 (43)
Median age in months (IQR)	14 (8, 24)	13 (8, 24)
Median length of hospital stay in days (IQR)	9 (6,13)	4 (2,6)
Median number of children in the household (IQR)	3 (2, 5)	3 (2, 4)
Caregiver		
Female, n (%)	476 (96)	224 (95)
Biological parent, n (%)	467 (95)	225 (95)
Mean age in years (SD)	30 (8.9)	29 (7.3)
Education level, n (%)		
None	63 (13)	20 (8)
Primary	300 (61)	136 (57)
Secondary	88 (18)	61 (26)
Tertiary	30 (6)	17 (7)
Main activity, n (%)		
Income activity	207 (42)	102 (43)
Non-income activity	287 (58)	135 (57)
Source of income, n (%)		
None	287 (58)	135 (57)
Paid salary	99 (20)	40 (17)
Own business	108 (22)	62 (26)
Median monthly individual income in USD (IQR)	0 (0, 39)	0 (0, 59)
Household characteristics		
Median monthly household income in USD (IQR)	69 (20, 147)	98 (39, 176)
Median household size (IQR)	5 (4, 8)	5 (4, 6)
Household asset tertiles, n (%)		
1 (poorest)	174 (35)	70 (30)
2	180 (35)	64 (27)
3 (least poor)	140 (28)	103 (44)
Access to health facility		
Main means of travel to hospital, n (%)		
Public motorised means	311 (63)	116 (49)
Private motorised means	33 (7)	15 (6)
Walking/bicycle/motorcycle/tuk-tuk	121 (25)	83 (35)
Ambulance	29 (6)	23 (10)

CSM, complicated severe malnutrition; USD, US dollars.

### Direct costs

Six participants reported unknown direct healthcare costs while all the participants reported direct non-health care costs. A total of 354 participants (48%) were accompanied by at least one person to hospital with known associated costs being available from 344 (97%) of these participants. The largest direct costs during hospitalisation were bed charges and food (table 2). Bed charges, food, drugs and diaper costs were significantly higher for CSM than non-CSM children. Kenyan sites had higher direct healthcare and non-healthcare costs than Ugandan sites, median US$22 vs US$0 and US$17 vs US$12, respectively (online supplemental etable 2). In Kenya, bed charges were the main cost driver while in Uganda, cost of food was the main driver. Costs also varied by subnational site (online supplemental efigure 1).

**Table 2:** Direct costs in US dollars during hospitalisation by nutritional status

Cost itemmedian (IQR)*Mean (SD)*	TotalN=731	CSMN=596	Non-CSMN=237	P value*
Direct healthcare costs				
Administration	1.96 (0.00, 2.94)*2.06* (*2.54*)	1.47 (0.00, 2.94)*2.07* (*2.74*)	1.96 (1.18, 2.94)*2.04* (*2.12*)	0.298
Bed charges	8.82 (1.47, 26.96)*17.52* (*23.56*)	14.71 (2.45, 30.88)*19.95* (*25.49*)	6.86 (0.98, 17.65)*16.63* (*18.19*)	<0.001
Drugs	0.00 (0.00,1.67)*2.90* (*8.88*)	0.00 (0.00, 2.78)*3.25* (*10.08*)	0.00 (0.00, 0.83)*2.24* (*8.88*)	0.029
Diagnostic tests	0.00 (0.00, 0.00)*1.92* (*9.32*)	0.00 (0.00, 0.00)*1.72* (*7.68*)	0.00 (0.00, 0.00)*2.29* (*11.83*)	0.003
Direct non-health care costs				
Travel	1.18 (0.69, 2.45)*2.35* (*4.96*)	1.39 (0.78, 2.45)*2.20* (*4.07*)	0.98 (0.69, 2.45)*2.67* (*6.43*)	0.211
Food	3.73 (0.00, 5.88)*5.09* (*7.19*)	4.41 (0.00, 7.78)*6.03* (*8.26*)	2.78 (0.00, 4.41)*3.28* (*3.79*)	<0.001
Diapers	2.94 (1.18, 5.88)*4.51* (*5.44*)	3.92 (1.96, 7.45)*5.70* (*6.04*)	1.47 (0.00, 2.94)*2.06* (*2.58*)	<0.001
Companion costs	4.02 (1.96, 12.25)*10.99* (*18.72*)	4.90 (1.96, 13.73)11.65 (19.49)	3.14 (1.96, 10.39)9.53 (16.87)	0.142
Other costs	3.53 (1.17, 23.28)18.45 (32.65)	3.68 (1.67, 24.51)19.68 (34.04)	2.94 (0.29, 21.08)15.98 (29.56)	0.067

For analysis and interpretation of significance test, we used median values due to the skewed nature of cost data. Mean values (in italics) are provided for informational purposes.

* Kruskal-Wallis tests between CSM and non-CSM children.

CSM, complicated severe malnutrition.

Median preadmission, hospitalisation and postdischarge direct costs were US$5, US$42 and US$8, respectively, overall: US$4, US$47 and US$8 for CSM children compared with US$5, US$33 and US$2 for non-CSM children (table 3 and online supplemental etable 3; p=0.60, <0.001, 0.01).

**Table 3:** Timing of direct and indirect costs of severe malnourished and non-severe malnourished children, 2018/2019 in US dollars

	Total direct costs		Days lost*		Total indirect costs		Total costs(direct and indirect costs)
	Mean(SD)	Median(IQR)	Median(IQR)	Mean(SD)	Median(IQR)	Mean(SD)	Median(IQR)
CSM children							
Preadmission	21.45(64.9)	4.43(1.38,15.34)	1(1,3)	3.71(10.76)	0(0,1.93)	24.67(68.4)	6.10 (1.96,20.59)
During hospitalisation	63.49(59.8)	47.39(23.44,82.35)	8(6,13)	11.25(28.2)	0(0,11.57)	75.28(77.8)	55.60(25.84,98.53)
Postdischarge	14.81(24.7)	7.84(2.94,15.32)	2(1,4)	2.34(6.58)	0(0,1.61)	16.97(25.8)	8.29(3.59,16.79)
TOTAL CSM	N/A	N/A	N/A	N/A	N/A	97.5 (105)	68.33(36.15,119.3)
Non-CSM children							
Preadmission	16.42(42.8)	5.39(1.96,13.82)	1(1,2)	3.60(13.20)	0(0,3.21)	19.50(48.1)	6.90 (2.35,16.67)
During hospitalisation	46.85(48.8)	33.14(16.67,58.82)	4(3,6)	7.29(18.2)	0(0,7.71)	57.85(76.6)	35.78(19.80,64.62)
Postdischarge†	1.67 (1.15)	1.96(1.18,2.16)	1(1,1)	0.94(1.33)	0(0,1.82)	2.61(1.41)	2.16(1.82,3.06)
Total non-CSM	N/A	N/A	N/A	N/A	N/A	66.9 (81)	44.31(23.34,75.98)

Total indirect costs were calculated by multiplying number of days lost by reported income for the caregivers. This did not include indirect costs for the companion and other carers at home.

* Caregiver’s time loss in days. Outpatient stay of less than 24 hours was assumed to be 1 day.

† Postdischarge for non-CSM children was from a small sample size (n=5). None had persons accompanying.

CSM, complicated severe malnutrition; N/A, not available.

A total of 214 (29%) caregivers received waivers for their direct hospitalisation costs, with similar proportions for CSM and non-CSM children (p=0.18). Only 5 (<1%) caregivers received full waivers. Out of those that received waivers, a total of 183 (86%) caregivers were waived more than half of their costs with only 13% in the poorest quintile and 52% in the least poor category receiving waivers.

## Indirect costs

Caregivers of CSM children lost a median of 8 working days (IQR: 6–13) during hospitalisation, compared with 4 (IQR: 3–6) for non-CSM caregivers (p<0.0001) (table 3). Productivity loss did not differ by country (online supplemental etable 2).

In some instances, caregivers requested other caretakers to look after other children or dependents at home while away (482/731 (66%). Overall, these caretakers lost a median of 7 days (IQR 4–11). Among the 482 caretakers, only 39 (0.08%) were paid or were to be paid for their time.

### Overall costs per child

Total costs were a median of US$60 (IQR US$32–US$107) (table 3). Total costs, costs during hospitalisation and postdischarge (p<0.001, p<0.001 and p=0.02, respectively), but not preadmission, were higher for CSM than non-CSM children.

### Coping mechanisms

**Figure 1:**
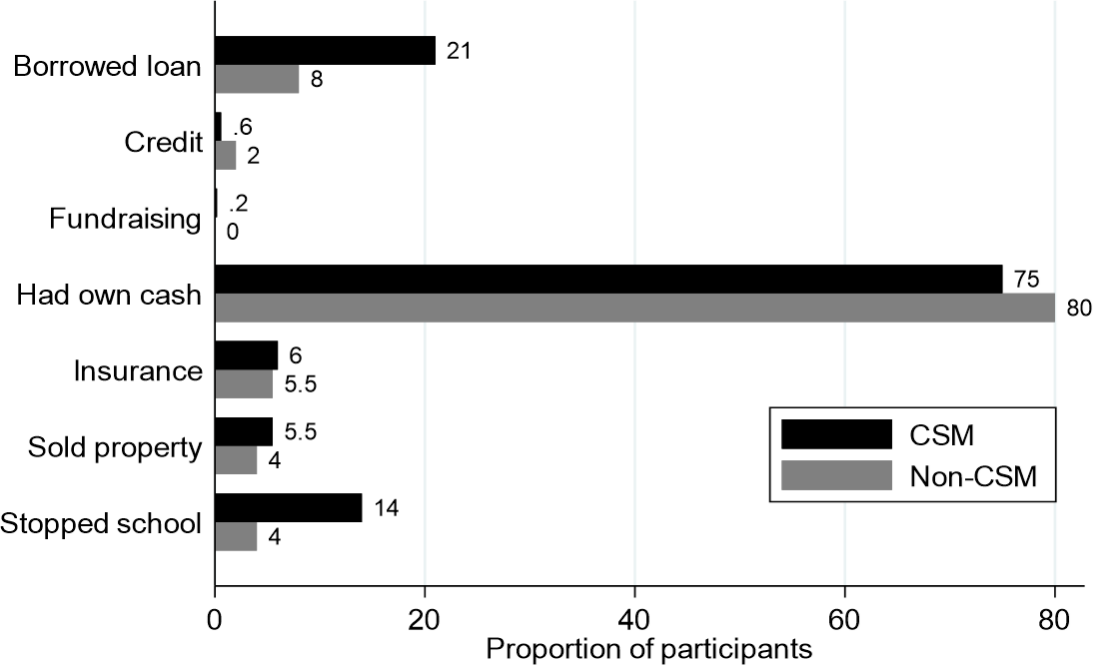
Coping mechanisms and sources of funds for incurred costs during hospitalisation 58% borrowed from neighbours and friends and 27% from family. Sold livestock, household item or farm produce. Stopped school indicates other children and dependents back at home that stopped going to school due to the sick child’s hospitalisation. CSM, complicated severe malnutrition.

Coping mechanisms included 124 (17%) borrowing from friends and family (21% vs 8% in CSM and non-CSM, p<0.001). A total of 78 (11%) households reported taking other children and dependents out of school (14% vs 4% in CSM and non-CSM: p<0.001). Assets were sold by 37 (5%) participants while only 43 (6%) had national health insurance (figure 1).

### Income and catastrophic expenditure

Overall, 324/731 (44%) caregivers could report household income and 60 (19%) indicated that they had no household income. Caregivers who could not report household income were mostly Kenya and had significantly lower individual income (median US$$0 (IQR US$0, US$25) vs US$2 (IQR US$0, US$59)) than those who could report household income (online supplemental etable 4). Children with CSM, other costs incurred and demographic characteristics were similar between those that did and did not report household income (online supplemental etable 4). Individual income, but not household income, was reported by 111 (15%).

**Figure 2:**
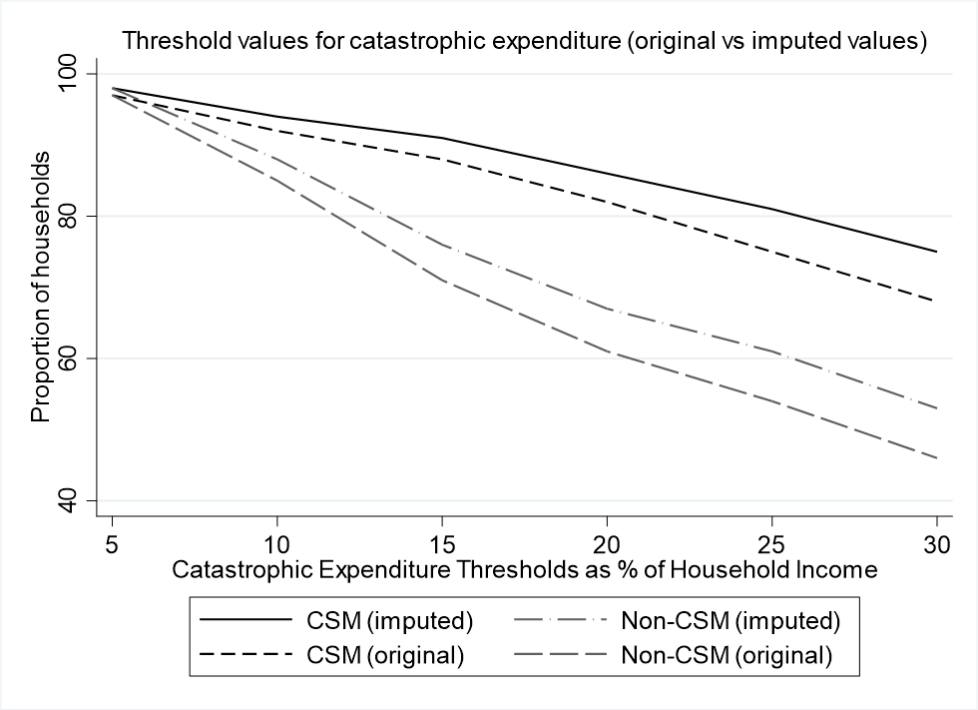
Catastrophic expenditure with sensitivity analysis of original values and imputed household income. CSM, complicated severe malnutrition.

Using individual income for household income for these participants, we included data from 375 (51%) participants in the primary analysis of catastrophic expenditure (252 CSM and 123 non-CSM). Catastrophic expenditure at 10% and 25% threshold was experienced by 94% vs 88% (p=0.105) and 81% vs 61% (p<0.001) for CSM and non-CSM households, respectively (figure 2). Using imputed values to include all participants suggested higher proportions of suffering catastrophic expenditure (figure 2).

### Sensitivity analyses

Costs due to productivity loss estimated using per capita; GDP per capita; expected minimum wage and informal workers’ wage for non-income earners were all higher than estimated from reported individual income (online supplemental etable 5).

For thresholds between 5% and 30%, the proportion of households experiencing catastrophic expenditure was significantly different between CSM and non-CSM for all thresholds above 10%. The pattern was similar using reported household income and imputed values (figure 2).

## Discussion

We found that in Kenya and Uganda, catastrophic healthcare expenditure associated with hospitalisation of acutely ill children was extremely common, affecting 92% and 74% of households at thresholds of 10% and 25% of monthly income depending on the presence of CSM. OOP costs were the main drivers, especially during hospitalisation, primarily from hospital bed charges in Kenya and from food in Uganda. Costs varied between the different hospitals in East Africa included in this study, but overall total direct and indirect costs of hospitalisation per child with CSM of US$56 were similar to previous studies of CSM^17^ and of other childhood illnesses (US$47–US$82 for malaria, US$54–US$99 for pneumonia and US$86 for diarrhoeal illness).^12 39^

Kenya and Uganda introduced free healthcare services to children under 5 years old in 2004 and 2001 respectively.^40 41^ OOP costs were much higher in Kenya (15-fold) than in Uganda, where these costs were minimal (drugs and diagnostic tests) or free (administration and bed charges). Bed charges in Kenyan hospitals are still high, despite the policy that under 5s are exempt from these charges.^42^ Non-healthcare costs such as transportation, food and diapers were an additional burden, with Kenyan caregivers spending twice as much on these items as Ugandan caregivers. In another Kenyan study, one-third of mothers reported using disposable diapers, 60% used both cloth and disposable diapers and only 4% used cloth diapers exclusively during hospitalisation.^43^

Inadequate access to resources is one of the main drivers of child undernutrition as outlined in the UNICEF framework^44^ and both direct and indirect effects on mortality were demonstrated in the CHAIN cohort.^13^ After discharge, a child returning to a household that has experienced catastrophic effects may contribute to cycles of illness and poverty.^45^ Interventions supporting financial protection such as subsidies, exemptions and fee waivers, together with socioeconomic support, such as cash transfer and educational programmes, may help mitigate these challenges. Provision of transport vouchers might reduce costs and improve access during the vulnerable postdischarge period.^4^ In Kenya, mobile banking and transport vouchers have improved access to maternal health services.^46^ Overall, health system changes to help provide food and diapers, and reduce or eliminate bed charges, and/or targeted cash or voucher interventions have potential to make a major impact on catastrophic healthcare spending among economically vulnerable families. Cost-effectiveness, whether this translates to reduced postdischarge mortality and assessment of unintended consequences, will require careful clinical trials.^47^,^50^

As previously reported, families used a variety of coping strategies including borrowing money from family and friends, mostly interest-free, showing the importance of social networks.^51^ Six per cent of participants indicated that they had access to health insurance and only one-third overall received waivers from the hospitals despite the availability of National Hospital Insurance Fund or Scheme in Kenya and Uganda respectively. There was a disparity in those that received waivers as it did not reach the most vulnerable. Health insurance subsidies should be extended to the extremely poor and provide financial protection.

The main strengths of this study are the use of three large cohorts of acutely ill children in multiple settings in East Africa, detailed data and sensitivity analysis to assess different scenarios for productivity losses and CHE thresholds. Limitations include smaller sample size for the postdischarge analysis in the non-CSM group. Therefore, we must be cautious when comparing statistical differences in this group. The study design meant that we enrolled a higher proportion with CSM than typically admitted, hence provided results split by CSM status. Cost data may have been subject to recall bias, especially costs incurred prior to hospitalisation and postdischarge costs. Unreported, under-reported or over-reporting of household income, expenditure and costs are a common phenomenon. This is likely to have biased the catastrophic expenditure analysis. We would have verified expenditure and costs using receipts or bills to improve accuracy but due to the lack of availability of these documents from all patients, we avoided acquiring these to avoid further bias of differential calculation of costs among the participants. Finally, individual and household income was collected at only one time point, and we were not able to ascertain the duration over which catastrophic costs were experienced by the families.

## Conclusions

A very large proportion of households of acutely ill children experienced catastrophic economic shock associated with hospitalisation. Interventions to alleviate the cost burden of hospitalisation, including health systems reforms, reducing or removing bed charges for carers of children under 5, providing a clearer framework of exemptions and waivers (particularly around hospitalisation-associated costs), provision of basic items (such as food and diapers) in hospital, improved access to insurance, social services and network support for families, and targeted cash transfer may have the potential to reduce the economic burden on families and improve child survival. Future interventions need to adopt societal perspective to reduce economic burden on families. However, such interventions need to be carefully designed to ensure that they are cost-effective, are acceptable and perceived to be beneficial to households/society, do not exacerbate inequities and that they reach those most in need.

## Supplementary material

10.1136/bmjph-2024-001173 online supplemental file 1

10.1136/bmjph-2024-001173 online supplemental file 2

## Data Availability

Data are available on reasonable request.
