# Supplementary material for: Catastrophic expenditure associated with childhood hospitalisation for acute illness in Kenya and Uganda: a cross-sectional study
Source: BMJ Public Health. 2025 Jan 16;3(1):e001173. doi: 10.1136/bmjph-2024-001173 (PMC11812878; doi:10.1136/bmjph-2024-001173)
Supplement: online supplemental file 1 [file bmjph-3-1-s001.pdf]

eTable 1: Description of study cohorts and sites included in costing analysis

Cohorts	Description of the parent studies	Sites included for costing study
A. FLACSAM trial (19)	A factorial randomized clinical trial assessing efficacy of different first line antimicrobials in children with severe malnutrition defined by WHO criteria using MUAC, weight-for-length and presence of kwashiorkor among children aged between 2 months and 13 years of age. Economic data were collected at discharge and 45 days after enrolment	Kilifi County Hospital; Coast General Hospital; Mbagathi Hospital; Mbale Regional Referral Hospital.
B. FLACSAM parallel cohort	A cohort of children aged between 2 months and 13 years of age without severe malnutrition, prospectively enrolled in parallel to the FLACSAM trial. Economic data were collected at discharge only as there was no follow-up in this cohort.	Kilifi County Hospital; Coast General Hospital; Mbagathi Hospital; Mbale Regional Referral Hospital.
C. CHAIN cohort[a] (13)	An observational study that enrolled children aged 2 to 23 months with and without severe malnutrition. Data were collected at hospital admission, discharge and 45 days after discharge.	Mulago National Referral Hospital; Migori County Referral Hospital.

FLACSAM: First Line Antimicrobials in Children with Complicated Severe Acute Malnutrition; CHAIN: The Childhood Acute Illness and Nutrition

[a]CHAIN cohort was conducted in nine sites across Africa and Asia. For this study, we selected participants in Kenya and Uganda only

eTable 2: Direct and indirect costs during hospitalisation by country of study

**Cost item,**<br>Median [IQR]<br>*Mean (SD) in italics*	**Total N=731**	**Kenya N=596**	**Uganda N=135**	**P-value***
**Direct health care and non-health care costs**				
Administration	1.96 [0.00,2.94]<br>*2.06 (2.54)*	2.94 [0.98,2.94]<br>*2.54 (2.60)*	0.00 [0.00,0.00]<br>*0.03 (0.30)*	<0.001
Bed charges	8.82 [1.47,26.96]<br>*17.52 (23.56)*	15.69 [4.41,30.88]<br>*21.63 (24.44)*	0.00 [0.00,0.00]<br>*0.13 (1.45)*	<0.001
Drugs	0.00 [0.00,1.67]<br>*2.90 (8.88)*	0.00 [0.00,2.25]<br>*3.35 (10.72)*	0.00 [0.00,1.39]<br>*1.10 (2.46)*	0.893
Diagnostic tests	0.00 [0.00,0.00]<br>*1.92 (9.32)*	0.00 [0.00,0.00]<br>2.18 (10.30)	0.00 [0.00,0.00]<br>0.87 (2.87)	0.763
Travel	1.18 [0.69,2.45]<br>*2.35 (4.96)*	1.18 [0.69,2.45]<br>*2.46 (5.42)*	1.39 [0.83,2.78]<br>*1.87 (1.85)*	0.967
Food	3.73 [0.00,5.88]<br>*5.09 (7.19)*	2.94 [0.00,4.41]<br>*3.98 (6.29)*	8.34 [3.33,12.99]<br>*9.74 (8.67)*	<0.001
Diapers	2.94 [1.18,5.88]<br>*4.51 (5.44)*	3.53 [1.91,6.23]<br>*4.92 (5.53)*	1.11 [0.00,3.89]<br>*2.74 (4.65)*	<0.001
Direct companion costs	4.01 [1.96,12.25]<br>*10.99 (18.72)*	3.92 [1.57,9.80]<br>8.86 (15.27)	11.67[5.56,26.67]<br>21.71 (28.56)	<0.001
Other costs	3.53 [1.17,23.28]<br>18.45 (32.65	4.90 [1.96,34.31]<br>*22.16 (35.07)*	0.97 [0.00,2.78]<br>*2.12 (4.46)*	<0.001
**Overall costs**				
Direct health care costs	16.67 [4.17,32.84]<br>*23.89 (30.41)*	22.01 [8.82,36.27]<br>28.83 (31.61)	0.00 [0.00,2.22]<br>2.33 (5.32)	<0.001
Direct non-health care costs	16.18 [8.81,35.84]<br>*29.27 (35.06)*	17.06 [9.61,41.91]<br>32.24 (37.49)	12.23 [6.39,22.23]<br>16.12 (15.58)	<0.001
Indirect costs	0 [0, 9.64]<br>*10.01 (25.57)*	0 [0,11.57]<br>*11.21 (27.58)*	0 [0,4.46]<br>*4.28 (10.11)*	0.204

**Kruskal-Wallis tests between median values.*
For analysis and interpretation of significance test, we used median values due to the skewed nature of cost data. Mean values (in italics) are provided for informational purposes.
IQR: Interquartile range, SD: Standard deviation

eTable 3: Timing of direct costs of severe malnourished and non-severe malnourished children, 2018/2019 in US dollars

	Direct health care costs		Direct non-healthcare costs		Direct companion costs		**Total direct costs**	
	Mean (SD)	*Median [IQR]*	*Mean (SD)*	*Median [IQR]*	*Mean (SD)*	*Median [IQR]*	***Mean (SD)***	***Median [IQR]***
**CSM children**								
Pre-admission	19.02 (62.8)	2.94 [0.00,13.24]	2.94 (6.29)	1.11 [0.00,2.76]	3.55 (11.7)	0.98 [0.19,3.04]	**21.45 (64.9)**	**4.43 [1.38,15.34]**
During hospitalisation	26.27 (32.5)	18.24 [4.71,36.27]	31.93 (36.7)	17.44 [10.19,39.61]	11.6 (19.5)	4.90 [1.96,13.73]	**63.49 (59.8)**	**47.39 [23.44,82.35]**
Post-discharge	7.58 (21.9)	0.78 [0.00,5.39]	5.51 (6.33)	2.94 [0.98,8.33]	6.46 (9.39)	2.94 [0.78,7.84]	**14.81 (24.7)**	**7.84 [2.94,15.32]**
**TOTAL CSM**	N/A	N/A	N/A	N/A	N/A	N/A	N/A	N/A
**Non-CSM children**								
Pre-admission	14.59 (41.5)	4.90 [0.78,11.76]	1.92 (3.77)	0.78 [0.00,1.96]	1.67 (4.30)	0.49 [0.00,1.47]	**16.42 (42.8)**	**5.39 [1.96,13.82]**
During hospitalisation	18.88 (24.9)	12.75 [3.33,23.53]	23.70 (30.8)	12.75 [7.22,26.76]	9.53 (16.9)	3.14 [1.96,10.39]	**46.85 (48.8)**	**33.14 [16.67,58.82]**
Post-discharge[a]	0.24 (0.53)	0 [0,0]	1.43 (1.14)	1.18 [0.98,1.96]	N/A	N/A	**1.67 (1.15)**	**1.96 [1.18,2.16]**
**TOTAL Non-CSM**	N/A	N/A	N/A	N/A	N/A	N/A	N/A	N/A

[a]Post-discharge for non-CSM children was from a small sample size (n=5). None had persons accompanying.
IQR: Interquartile range, SD: Standard deviation, CSM: Complicated Severe malnutrition, Non-CSM: Non-complicated severe malnutrition

eTable 4: Participant characteristics of those that reported household income compared to those that did not report

	**Participants that reported household income N=324**	**Participants that did not report household income N=407**	**P-value**
Study group, n (%)			
CSM	217 (67)	277 (68)	0.814
Non-CSM	107 (33)	130 (32)	0.870
Country, n (%)			
Kenya	237 (73)	359 (88)	<0.001
Uganda	87 (27)	48 (12)	0.043
Main activity, n (%)			
Income activity	158 (49)	151 (37)	0.033
Non-Income activity	166 (51)	256 (63)	0.015
Median monthly individual income in USD [IQR]	1.96 [0.00,58.82]	0.00 [0.00,25.01]	<0.001
Household asset tertiles, n (%)			
1 (Poorest)	100 (31)	144 (35)	0.251
2	118 (36)	126 (31)	
3 (Least Poor)	106 (33)	137 (34)	
Median direct costs in USD [IQR]	42.89 [21.19,75.34]	41.47 [21.47,78.43]	0.672
Median indirect costs in USD [IQR]	0.11 [0.00,13.50]	0.00 [0.00,5.79]	<0.001
Median overall costs in USD [IQR]	49.26 [25.29,85.72]	44.61 [23.44,81.86]	0.334

USD: United States dollars, IQR: Interquartile range, CSM: Severe malnutrition, Non-CSM: Non-severe malnutrition

eTable 5: Sensitivity analysis of indirect costs to the caregivers during hospitalization using different estimates of daily wage rate

Estimation approaches	Daily Rate in USD	Daily Rate in USD	Indirect cost Median [IQR] *Mean (SD)*	Indirect cost Median [IQR] *Mean (SD)*
	**Kenya**	**Uganda**	**CSM**	**Non-CSM**
GNI per capita[a]	12.62	6.11	97.76 [75.72,151.44] *118.75 (88.1)*	50.48 [25.24,75.72] *60.72 (59.7)*
GDP per capita[b]	12.82	6.28	100.48 [76.92,153.84] *120.79 (89.4)*	51.28 [25.64,76.92] *61.74 (60.7)*
Minimum wage[c]	2.25	1.20	18.00 [13.50,27.00] *21.42 (15.7)*	9.00 [4.50,13.50] *10.91 (10.7)*
Forgone wage substitute for non-income earners[d]	5.73	3.82	34.38 [15.43,57.30] *42.75 (39.8)*	17.19 [7.64,28.65] *23.29 (23.9)*
Reported individual income (reference)			0 [0,11.57] *11.25 (28.2)*	0 [0,7.71] *7.29(18.2)*

USD: United States dollars, IQR: Interquartile range, SD: Standard deviation, SM: Severe malnutrition, Non-SM: Non-severe malnutrition, GNI: Gross National Income, GDP: Gross Domestic Product

[a]GNI Per Capita, PPP (Current International, 2018/2019) according to World Bank Development Indicators

[b]GDP Per Capita, PPP (Current International, 2018/2019) according to World Bank Development Indicators

[c]Cost of Living Survey 2018/2019, Wage Indicator: https://costofliving.wageindicator.org/

[d]Average wage rate for informal workers in Kenya: https://epzakenya.com/wp-content/uploads/2020/09/EPZ-Annual-Performance-Report-year-2019.pdf and in Uganda from Cost of Living Survey 2019, Wage Indicator: https://costofliving.wageindicator.org/
