# Supplementary material for: Catastrophic expenditure associated with childhood hospitalisation for acute illness in Kenya and Uganda: a cross-sectional study
Source: BMJ Public Health. 2025 Jan 16;3(1):e001173. doi: 10.1136/bmjph-2024-001173 (PMC11812878; doi:10.1136/bmjph-2024-001173)
Supplement: online supplemental file 2 [file bmjph-3-1-s002.pdf]

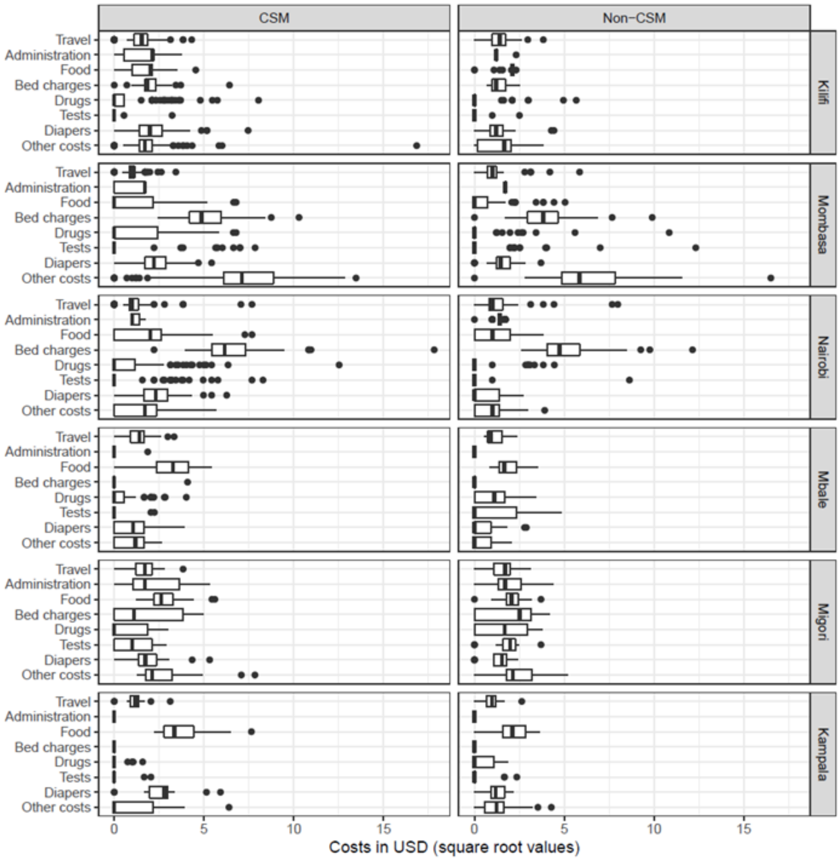

eFigure 1: Directs costs incurred during hospitalisation by study site and nutritional status

The costs were transformed to the square root value for purposes of better visualization of the cost drivers and differences between cost categories. This was the most reasonable transformation approach based on our data and due to the high zero values in the cost data.
